# Efficacy of the Combination of Systemic Sequential Therapy and Locoregional Therapy in the Long-Term Survival of Patients with BCLC Stage C Hepatocellular Carcinoma

**DOI:** 10.3390/cancers15153789

**Published:** 2023-07-26

**Authors:** Yusuke Kawamura, Norio Akuta, Junichi Shindoh, Masaru Matsumura, Satoshi Okubo, Licht Tominaga, Shunichiro Fujiyama, Tetsuya Hosaka, Satoshi Saitoh, Hitomi Sezaki, Fumitaka Suzuki, Yoshiyuki Suzuki, Kenji Ikeda, Yasuji Arase, Masaji Hashimoto, Takuyo Kozuka, Hiromitsu Kumada

**Affiliations:** 1 Department of Hepatology Toranomon Hospital 2-2-2, Toranomon, Minato-ku, Tokyo 105-8470, Japan; akuta-gi@umin.ac.jp (N.A.); shunichiro-fujiyama@toranomon.gr.jp (S.F.); hosa-p@toranomon.gr.jp (T.H.); sa3110@f2.dion.ne.jp (S.S.); hitomis@mx1.harmonix.ne.jp (H.S.); fumitakas@toranomon.gr.jp (F.S.); suzunari@interlink.or.jp (Y.S.); ikedakenji@tora.email.ne.jp (K.I.); es9y-ars@asahi-net.or.jp (Y.A.); kumahiro@toranomon.gr.jp (H.K.); 2 Okinaka Memorial Institute for Medical Research, Toranomon Hospital, Tokyo 105-8470, Japan; shindou-tky@umin.ac.jp (J.S.); teamma22n@gmail.com (M.M.); sokubo@toranomon.gr.jp (S.O.); licht@toranomon.gr.jp (L.T.); masaji.hashimoto@gmail.com (M.H.); kozuka@toranomon.gr.jp (T.K.); 3 Hepatobiliary-Pancreatic Surgery Division, Department of Gastroenterological Surgery, Toranomon Hospital, Tokyo 105-8470, Japan; 4 Radiation Oncology Division, Department of Radiology, Toranomon Hospital, Tokyo 105-8470, Japan

**Keywords:** hepatocellular carcinoma, lenvatinib, atezolizumab plus bevacizumab, systemic therapy, locoregional treatment, combination therapy

## Abstract

**Simple Summary:**

The Barcelona clinic liver cancer (BCLC) system is used widely for staging hepatocellular carcinomas (HCCs). However, it is questionable that for patients classified as BCLC stage C, control of intrahepatic targets using various treatment procedures is not the main topic of discussion, whereas the importance of intrahepatic tumor control in patients with extrahepatic tumor spread is reviewed. Therefore, this study analyzed the data of 64 consecutive BCLC stage C patients with intrahepatic target nodules who received systemic therapy and evaluated the efficacy of the combined use of systemic sequential therapy, including more than two agents, and locoregional treatment administered after initiation of systemic therapy. We showed that the combined use of systemic sequential therapy of more than two agents and locoregional-treatment improved overall survival in BCLC stage C HCC patients with intrahepatic target nodules who had previously received systemic therapy-based treatment.

**Abstract:**

Background: The aim of this study was to evaluate the clinical impact of a combination of systemic sequential therapy and locoregional therapy on the long-term survival of patients with Barcelona Clinic Liver Cancer (BCLC) stage C hepatocellular carcinoma (HCC). Methods: Sixty-four consecutive patients with intrahepatic target nodules who had initially received systemic therapy (lenvatinib and atezolizumab plus bevacizumab) were reviewed. The clinical impact of the combined use of systemic sequential therapy and locoregional therapy was evaluated by determining overall survival (OS). The combined use of systemic sequential therapy with more than two agents and locoregional treatment was defined as multidisciplinary combination therapy (MCT), while only systemic sequential therapy and repeated locoregional-treatment was defined as a single treatment procedure (STP). Results: R0 resection, MCT, and STP resulted in significantly better OS compared with no additional treatment (median OS, not reached vs. 18.2 months and 12.6 vs. 8.1 months, respectively; *p* = 0.002). Multivariate analysis confirmed that the use of R0 resection and MCT were associated with better OS (hazard ratio [HR]; 0.053, *p* = 0.006 and 0.189, *p* < 0.001, respectively) compared with that for STP (HR; 0.279, *p* = 0.003). Conclusions: MCT is may effective in patients with BCLC stage C HCC and intrahepatic target nodules who have previously received systemic therapy-based treatment.

## 1. Introduction

Hepatocellular carcinoma (HCC) is the most common form of liver cancer, which in turn is the third most common form of cancer [1]. The Barcelona Clinic Liver Cancer (BCLC) algorithm is used widely for staging HCCs [2,3,4], with current treatment strategies dependent on the results. For advanced-stage HCC (BCLC stage C), systemic therapy is recommended as first-line to later-line treatment. Atezolizumab plus bevacizumab (Atezo/Bev) is the recommended first-line combination therapy (immune checkpoint inhibitor [ICI] and a molecularly targeted agent [MTA]) for treatment of unresectable, advanced-stage HCC. Approximately one-third of HCC patients achieve an objective response with Atezo/Bev [5]. Similarly, lenvatinib can be administered as first-line treatment for advanced-stage HCC, especially in patients in whom Atezo/Bev is not indicated. In fact, systemic therapy is the definitive treatment strategy for advanced-stage HCC. However, it is questionable why control of intrahepatic targets in patients with BCLC stage C HCC using various treatment procedures is not the main topic of discussion. In contrast, the importance of intrahepatic tumor control in patients with extrahepatic tumor spread is reviewed [6,7,8,9,10,11,12]. Since the introduction of lenvatinib, encouraging results have been reported [13,14] on its highly synergistic effect with transarterial chemoembolization (TACE) [15,16,17,18] based on anti-tumor vessel effects [19] and high treatment efficacy in patients with oncologically aggressive HCC [20,21,22]. Recently, we carried out a study of lenvatinib-based treatment and showed that this treatment was effective in patients with advanced-stage HCC and intrahepatic target nodules [23]. BCLC intermediate-stage disease is quite heterogeneous and can be further subclassified using the Up-to-7 criteria [24] or Child-Pugh score [25]. A recent report suggested that TACE is preferred for patients with tumors within the Up-to-7 criteria who have good liver function [26]. In addition, several clinical trials [27,28,29] and the current AASLD guidelines [30] have reported that upfront molecularly targeted therapy followed by TACE is a useful treatment option in patients with high tumor burden beyond the Up-to-7 criteria.

However, the efficacy of the combined use of systemic sequential therapy of more than two agents and locoregional treatment (LT) after initiation of lenvatinib or Atezo/Bev remains unclear. Therefore, the current study evaluated the efficacy of the combined use of systemic sequential therapy of more than two agents and LT following initiation of lenvatinib or Atezo/Bev in patients with BCLC stage C HCC and an intrahepatic target lesion.

## 2. Materials and Methods

### 2.1. Study Population

Between October 2010 and December 2022, 149 consecutive patients received systemic lenvatinib (109) and Atezo/Bev (40) as first-line treatment for unresectable HCC, 64 patients were selected based on the following inclusion criteria: (1) dynamic-computed tomography (CT) or a magnetic resonance imaging (MRI) study performed within 1 month prior to initiation of lenvatinib or Atezo/Bev; (2) a tumor with hyperenhancement in the dynamic study; (3) Child-Pugh class A liver function at the time of initiation of lenvatinib or Atezo/Bev therapy; (4) BCLC stage C tumor(s); (5) unresectable HCC with the patient not wanting to undergo local ablation or chemoembolization therapy for various reasons (i.e., tumor size, number and location, extrahepatic spread, TACE refractoriness, or various complications); (6) no treatment history of systemic chemotherapy with immune checkpoint inhibitors and molecular targeted agents (e.g., sorafenib, lenvatinib and Atezo/Bev); (7) at least one measurable target nodule in the liver; and (8) an observation period of ≥4 weeks. All the procedures were carried out in accordance with the ethical standards of the responsible committees on human experimentation (institutional and national) and the criteria of the 1975 Helsinki Declaration. The study was approved by the Institutional Review Board of our hospital (protocol number 1438-H/B).

### 2.2. Diagnosis of HCC

Analysis of dynamic CT or magnetic resonance imaging (MRI) images was used to diagnose HCC. A nodule was diagnosed as HCC if it showed hyperattenuation in the arterial phase and washout in the portal or delayed phase on dynamic imaging.

### 2.3. Lenvatinib and Atezo/Bev Treatment and Assessment of Adverse Events

The majority of patients with an HCC nodule received oral administration of lenvatinib (Lenvima^®^, Eisai, Tokyo, Japan) at a dose of 8 mg/day for those with a bodyweight < 60 kg or 12 mg/day for those weighing ≥ 60 kg. The patients also received intravenous administration of atezolizumab (Tecentriq^®^, F. Hoffmann–La Roche Ltd., Basel, Switzerland/Genentech Inc., South San Francisco, CA, USA) (1200 mg) and bevacizumab (Avastin^®^; Genentech Inc., South San Francisco, CA, USA) (15 mg/kg) every three weeks. The occurrence of unacceptable or serious adverse events (AEs) or significant clinical tumor progression led to treatment being discontinued. Based on the dosing guidelines for lenvatinib and Atezo/Bev, the dose of lenvatinib was reduced, or treatment was discontinued if a patient developed a severe AE (i.e., ≥grade 3) or any unacceptable grade 2 drug-related AE. The National Cancer Institute’s Common Terminology Criteria for Adverse Events (CTCAE), version 4.0 [31], was used to assess the severity of the AEs. 

### 2.4. Definition of the Subsequent Treatment Procedure

For analysis, the types of subsequent treatments were stratified into the following three patterns: (1) single treatment procedure (STP), defined as a drug sequence or single drug and locoregional treatment; (2) multidisciplinary combination therapy (MCT) defined as more than two drug sequences combined with the use of locoregional treatment; and (3) R0 resection, defined as a patient who received curative surgical resection during the course of treatment, with or without tumor recurrence after surgery.

### 2.5. Treatment Protocol for Subsequent TACE and TAI during Lenvatinib Treatment

TACE alone or lenvatinib-TACE sequential therapy was used in patients who subsequently received TACE during their treatment course. Based on the condition of the tumor, these therapies were performed using a schedule and/or on-demand strategy. In patients with progressive disease (PD), the decision to continue the administration of lenvatinib was based on the measurement of liver function following TACE therapy and the physician’s judgment. TACE in both treatment groups involved intra-arterial injection of lipiodol plus either warmed miriplatin (Miripla^®^, Sumitomo Dainippon Pharma Co., Ltd., Osaka, Japan), cisplatin (IA-call^®^, Nippon Kayaku, Tokyo, Japan), or epirubicin (Farmorubicin^®^, Pfizer, Tokyo, Japan). This was followed by injection of 1 mm of gelatin particles (Gelpart^®^, Nippon Kayaku) mixed with contrast agent into the target blood vessel until complete obstruction was achieved in the tumor-feeding branch. In patients who received miriplatin, the injector containing a miriplatin/lipiodol suspension and sterilized physiological saline was placed in a container and warmed to 60 °C, followed by an injection of miriplatin (60 mg) suspended in 3.0 mL of lipiodol with the dose of miriplatin ranging from 50 to 100 mg. In patients receiving cisplatin, 100 mg cisplatin was dissolved in 70 mL saline, with the cisplatin and lipiodol solutions then divided into 7 to 10 parts. Aliquots of 7–10 mL of the cisplatin solution and 0.5–1 mL of lipiodol were then alternately and repeatedly infused, with the injected doses in each patient ranging between 60 and 100 mg for cisplatin and 3–5 mL for lipiodol. For patients who received epirubicin, the agent was suspended in 2–5 mL of lipiodol to prepare the contrast material containing 1/2 to 1/3 lipiodol or was loaded into drug-eluting beads (DC Beads^TM^, Boston Scientific, Marlborough, MA, USA) at a dose of 18–40 mg epirubicin per patient. All patients receiving lenvatinib-TACE sequential therapy were administered miriplatin or epirubicin as the first TACE procedure after receiving lenvatinib. The selection of the three anticancer agents was decided by the investigators, with on-demand TACE repeated until the occurrence of either treatment failure due to major progressive vascular invasion, the disappearance of the tumor-feeding artery, or deterioration in hepatic function. 

In addition, several patients in the cohort received Lenvatinib-Transhepatic Arterial Cisplatin infusion sequential Therapy (referred to as “L-TACT”) during their course of treatment. In the L-TACT regimen, lenvatinib was administered 7–14 days prior to TAI, and arterial CDDP infusion was performed under continuous lenvatinib administration.

### 2.6. Evaluation of Treatment Response

The Response Evaluation Criteria in Solid Tumours version 1.1 (RECIST ver.1.1) [32] was used to assess the response to treatment. An expert hepatologist (Y. Kawamura) and expert hepatobiliary surgeon (J. Shindoh), blinded to the clinical data, independently assessed the best tumor response over 2–12 weeks, with disagreements resolved by consensus review involving an additional investigator (K. Ikeda).

### 2.7. Definition of TACE Failure

CT or MRI was used to evaluate TACE failure after 1–3 months. Failure was defined as an insufficient response after ≥2 consecutive TACE procedures, even in patients in whom the chemotherapeutic agent had been changed and/or the feeding artery was redetermined, while TACE failure/refractoriness was defined as the appearance of a higher number of lesions in the liver than that recorded at the previous TACE procedure (other than the nodule being treated) [33].

### 2.8. Decision Process Regarding the Timing and Method of Subsequent Treatment

The timing and most appropriate additional subsequent treatment in patients with either a good response to treatment or progressive disease seen on imaging was discussed at a weekly multidisciplinary conference based on each patient’s tumor and liver status.

### 2.9. Assessment of Hepatic Functional Reserve

The hepatic functional reserve was assessed using the Child-Pugh classification [25] and the modified ALBI grade [34], based on the ALBI score, calculated from serum albumin and total bilirubin concentrations using the following formula: [ALBI score = (log10 bilirubin [µmol/L] × 0.66) + (albumin [g/L] × −0.085)], defined by the following cut-off values: ≤−2.60 = Grade 1; >−2.60 to ≤−2.27 = Grade 2a; >−2.27 to ≤−1.39 = Grade 2b; and >−1.39 = Grade 3 [35].

### 2.10. Follow-Up Protocol

After initiation of systemic therapy, the patients were evaluated every 1–3 weeks by physicians using biochemical and urine tests, in addition to dynamic CT or MRI, to assess early treatment responses during the 2–12 week period. Dynamic CT or MRI was performed every 1–3 months after the initial assessment of the best response.

### 2.11. Statistical Analysis

IBM SPSS software (ver. 29.0 SPSS Inc., Chicago, IL, USA) was used to perform the statistical analyses. The data were expressed as the median and range, with differences in the background features between each parameter analyzed by Fisher’s exact test and Kruskal–Wallis test. The significance of trends in changes in serum LDH levels during treatment with lenvatinib was evaluated using Friedman’s test. *p*-values < 0.05 were considered to indicate statistical significance. Overall survival (OS) after the introduction of systemic therapy was estimated using the Kaplan–Meier method, with the values compared using log-rank testing. 

Multivariate analysis using a Cox proportional hazard model was used to identify factors associated with OS after the initiation of systemic therapy. This analysis included both pre-treatment parameters and subsequent treatment as possible factors for intervention. All the factors showing a marginally significant association with OS (*p* < 0.15) in the univariate analysis were entered into a stepwise Cox regression analysis, with significant variables selected by the stepwise method. A two-tailed *p*-value < 0.05 was considered statistically significant.

## 3. Results

### 3.1. Overview

As shown in Table 1, the median age of the study population was 73.5 years, and 51 (80%) of the patients were male. The median size of the largest tumor was 46.0 mm (range 11–175 mm). In the study cohort, 39 patients (61%) had macrovascular invasion, and 38 patients (59%) had extrahepatic spread. Twenty-nine patients (45%) had a TACE failure/refractoriness status, while 43 patients had died by the time of database lock (1 February 2023). The median observation period was 11.2 months.

### 3.2. Treatment Response after Initiation of Initial Systemic Therapy

Evaluation of the best treatment response assessed by RECIST ver. 1.1 showed an objective response rate (ORR) of 29.7%. 

### 3.3. Impact of General Landmark Predictive Factors for Overall Survival of Patients with BCLC Stage C HCC

**Figure 1:**
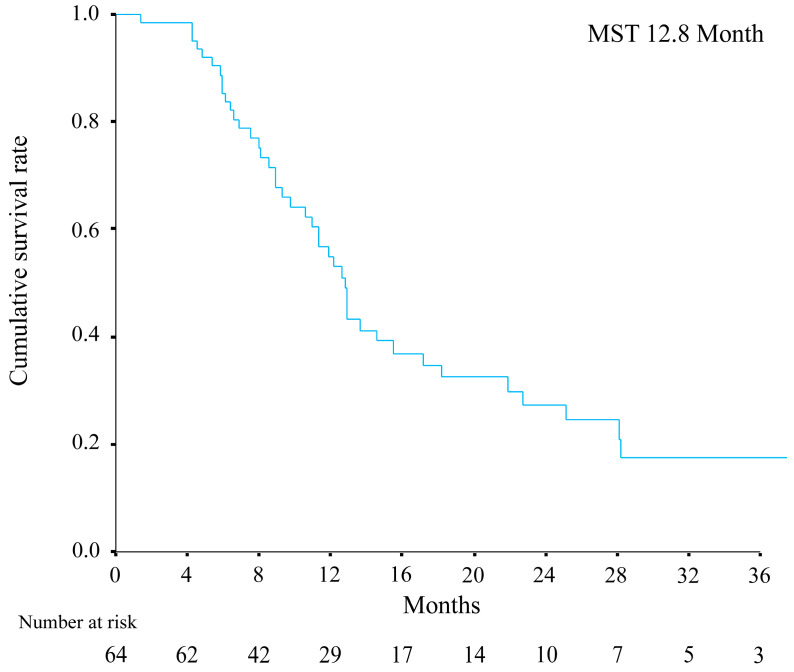
Overall survival outcomes of the HCC patients treated with systemic therapy.

Figure 1 shows the survival outcomes of patients with BCLC stage C HCC treated with systemic therapy. The median OS was 12.8 months.

**Figure 2:**
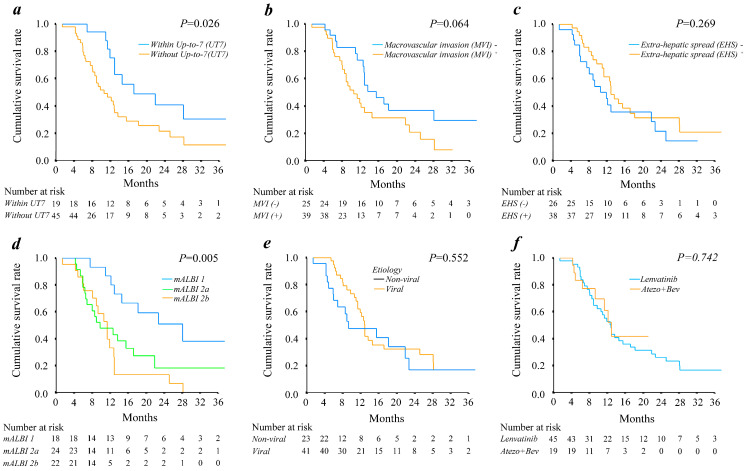
Overall survival outcomes of the HCC patients treated with systemic therapy grouped by (**a**) tumor burden (estimated using the Up-to-7 criteria), (**b**) presence of macrovascular invasion, (**c**) presence of extrahepatic spread, (**d**) mALBI grade, (**e**) liver disease etiology, and (**f**) systemic therapy type. UT7—up-to-7; MVI—microvascular invasion; EHS—extrahepatic spread; mALBI—modified albumin-bilirubin.

Figure 2 shows the impact of general HCC landmarks and predictive factors for OS. A significantly worse OS was associated with the presence of high tumor burden (exceeding the Up-to-7 criteria, *p* = 0.026; Figure 2a) and relatively worse residual liver function (vs. mALBI grade 1, *p* = 0.005; Figure 2d). Macrovascular invasion (Figure 2b, *p* = 0.064) also showed a tendency for poor OS. In contrast, no significant differences in OS were observed for the presence of extrahepatic spread (*p* = 0.269; Figure 2c), type of etiology (*p* = 0.552; Figure 2e), and treatment type of initial systemic therapy (*p* = 0.742; Figure 2f). 

**Figure 3:**
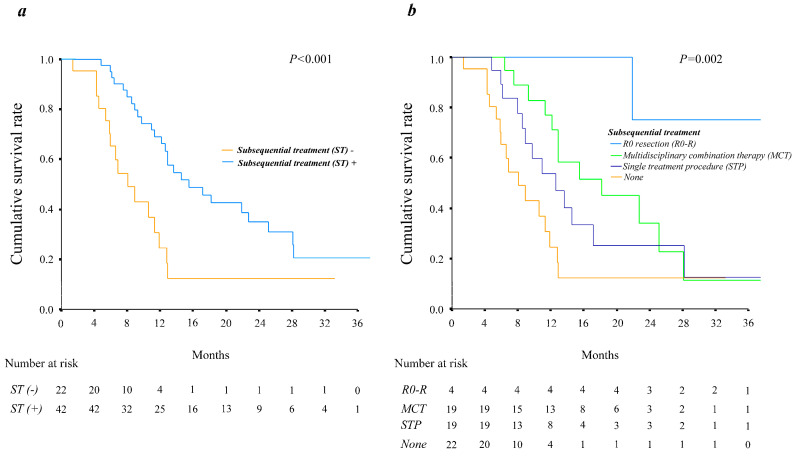
Overall survival curves of the HCC patients treated with systemic therapy, grouped by subsequent treatment during the treatment period. (**a**) Grouped by with or without subsequent treatment and (**b**) by type of subsequent treatment.

In addition, subsequent treatment during the treatment period resulted in significantly better survival (*p* < 0.001) (Figure 3a). A history of MCT and R0 resection during the course of treatment was also associated with significantly better OS (*p* = 0.002) (Figure 3b). 

### 3.4. Predictors of Overall Survival after Initiation of Systemic Therapy in Patients with BCLC Stage C HCC

Table 2 summarizes the results of the multivariate analysis for OS in patients treated by systemic therapy to BCLC stage C HCC. Of the 13 tested variables, exceeding the Up-to7 criteria (HR, 3.040; 95% CI, 1.291–7.155; *p* = 0.011), AFP level (HR, 1.003, 95% CI, 1.000–1.006, *p* = 0.023), and des-γ-carboxyprothrombin (DCP) level (HR, 1.001; 95% CI, 1.000–1.002; *p* = 0.006) were associated significantly with poor OS. In contrast, subsequent therapy (STP, MCT, and R0) was associated with better OS (HR, 0.279; 95% CI, 0.120–0.647; *p* = 0.003, HR, 0.189; 95% CI, 0.078–0.454; *p* < 0.001, and HR, 0.053, 95% CI, 0.006–0.440; *p* = 0.006, respectively). 

**Figure 4:**
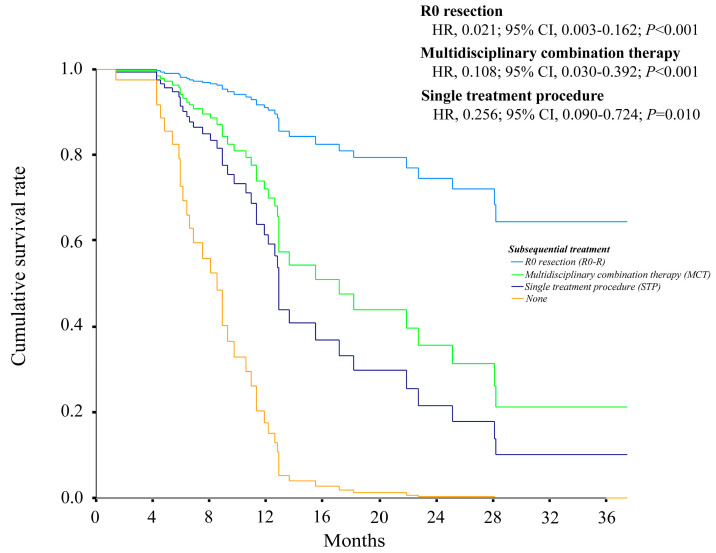
Adjusted overall survival curves of the HCC patients treated with systemic therapy, grouped according to the type of subsequent treatment selected during the treatment period. HR—hazard ratio; CI—confidence interval.

As shown in Figure 4, there were clear differences in the adjusted OS curves according to the subsequent treatment selected during systemic therapy.

### 3.5. The Relationship between Overall Survival and Various Types of Subsequent Treatments with or without Macrovascular Invasion

**Figure 5:**
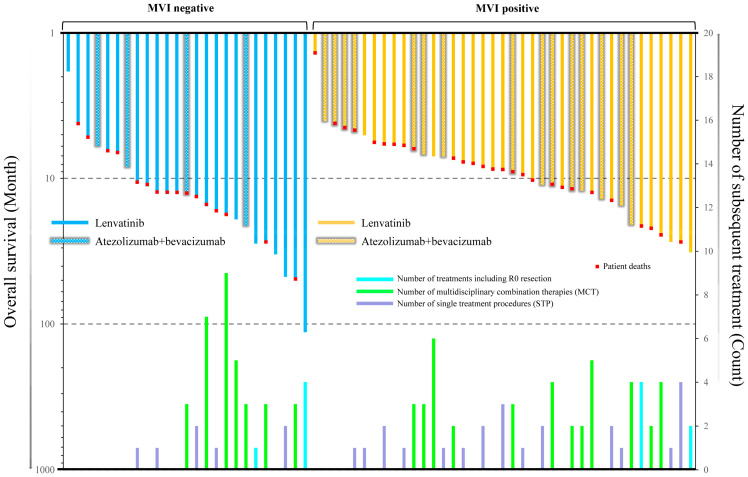
Relationship between overall survival and the types of subsequent treatment and number of treatments in patients with BCLC stage C HCC, grouped by MVI status. MVI—macrovascular invasion.

As shown in Figure 5, in order to achieve long-term survival of patients with BCLC stage C HCC, MCT was an important procedure whether or not the patient was MVI positive. Moreover, patients who received an R0 resection showed outstanding treatment outcomes.

### 3.6. Clinical Features of Patients with HCC Treated with Systemic Therapy Who Received or Did Not Receive Subsequent Treatment

As shown in Table 3, with the exception of patient age, no significant differences in patient or tumor characteristics were observed between those who received or did not receive subsequent treatment procedures. However, liver function and tumor burden tended to be better in patients who received an R0 resection. 

## 4. Discussion

This study evaluated the efficacy of the combined use of systemic sequential therapy of more than two agents and LT therapy following the administration of lenvatinib or Atezo/Bev in patients with BCLC stage C HCC and an intrahepatic target lesion. Our analyses showed that the presence of extrahepatic spread did not have a significant clinical impact on the OS of patients with BCLC stage C HCC treated with systemic therapy, a finding similar to that reported in previous studies [6,7,8,9,10,11,12]. In contrast, macrovascular invasion was associated with a poor prognosis in this patient population, indicating that invasion rather than extrahepatic spread is a more important prognostic factor in patients with BCLC stage C HCC. We also observed that residual liver function estimated by mALBI grade had a marked impact on OS.

Management of patients with an intrahepatic HCC is extremely important for prolonging OS. This was evident by our finding that a high tumor burden (e.g., exceeding the up-to-7 criteria) was also a significant prognostic factor. We also showed that subsequent treatment during systemic therapy, regardless of an R0 resection, resulted in MCT being associated with significantly better prognoses of patients. Multivariate analysis also showed that MCT was associated significantly with better OS. The number of R0 resections a patient can receive is limited by tumor and liver conditions. On the other hand, MCT may be used in more patients as a consequence of the foresight and enthusiasm of physicians. Therefore, it is important to focus on the importance of MCT during the treatment course of patients with BCLC stage C HCC.

**Figure 6:**
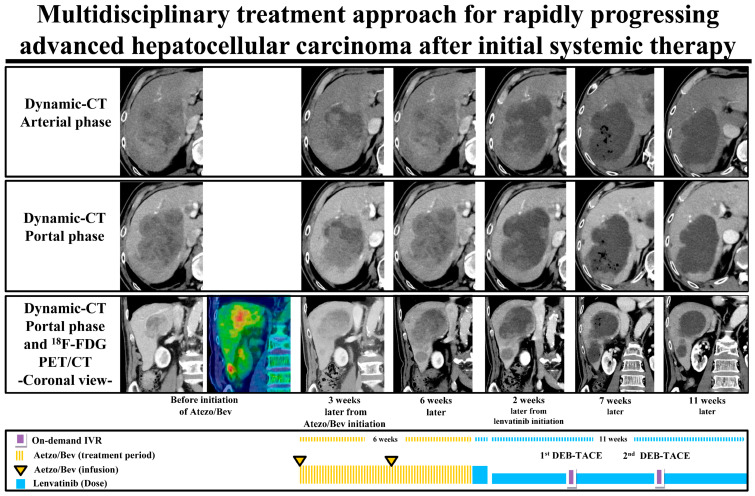
The flow of a multidisciplinary treatment approach for a patient with rapidly progressing advanced HCC after initial systemic therapy. The patient showed rapid progression of HCC after first-line Atezo/Bev therapy and received lenvatinib as second-line treatment. After initiation of lenvatinib, the tumor showed extensive necrosis and good tumor control in combination with subsequent DEB-TACE. Atezo/Bev—atezolizumab plus bevacizumab; DEB—drug-eluting beads; ^18^F-FDG-PET/CT—^18^F-fluorodeoxyglucose positron emission tomography/computed tomography; TACE—transarterial chemoembolization.

Notably, this case showed a marked tumor necrolytic effect with LDH increasing after the introduction of lenvatinib as a second-line treatment after the Atezo/Bev PD state (Figure 6). 

**Figure 7:**
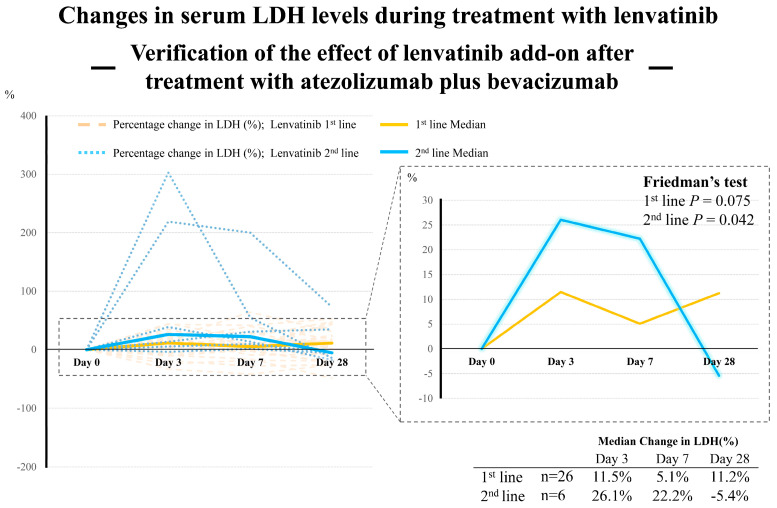
Verification of the efficacy of lenvatinib added after treatment with atezolizumab plus bevacizumab, based on LDH trends during the course of treatment.

As in previous reports [36,37], repeat administration of levatinib after diagnosis of disease progression during immunotherapy has the potential to affect disease control in some patients. However, the mechanism of this effect remains unclear. Therefore, we focused on this clinical question and performed additional research on the limited number of cases that had serum LDH levels evaluated using the estimated volume of tumor necrosis measured at days 3, 7, and 28 during first-line lenvatinib and second-line Atezo/Bev therapy. Figure 7 shows changes in serum LDH levels during the course of treatment in 26 and 6 patients who received first- and second-line lenvatinib therapy, respectively.

Initially, first-line lenvatinib therapy showed a marginally significant trend for changes in LDH levels. An even more notable result was that second-line lenvatinib therapy after Atezo/Bev showed a significant trend of increased LDH levels. As shown in Figure 7, the rate of change in LDH levels was markedly high on days 3 and 7, with a rapid decrease on day 28. These results may reflect the high anti-tumor effect of lenvatinib following immune therapy. However, these results were derived from a small number of cases. Therefore, we consider it necessary to perform additional studies on a larger number of patients in the future. 

As shown in Figure 3a,b, all subsequent treatments had significant positive effects on OS, especially those with a curative intent such as an R0 resection and MCT. 

In our study, we used multivariate analysis to identify factors that predicted OS, including subsequent treatments in systemic therapy. While this analysis should only be performed using pretreatment data, there is evidence that the majority of patients receiving systemic therapies develop disease progression relatively early during the course of treatment. This makes it necessary to also consider various subsequent treatments in patients who had sufficient residual liver function and were able to receive either RFA, surgical resection, radiotherapy, other immune checkpoint inhibitors, molecularly targeted agents, or TACE with or without systemic therapies (e.g., lenvatinib-TACE sequential therapy). As shown in Figure 4, the adjusted OS curves also showed a clear difference when systemic therapies based on MCT were used during the treatment period.

**Figure 8:**
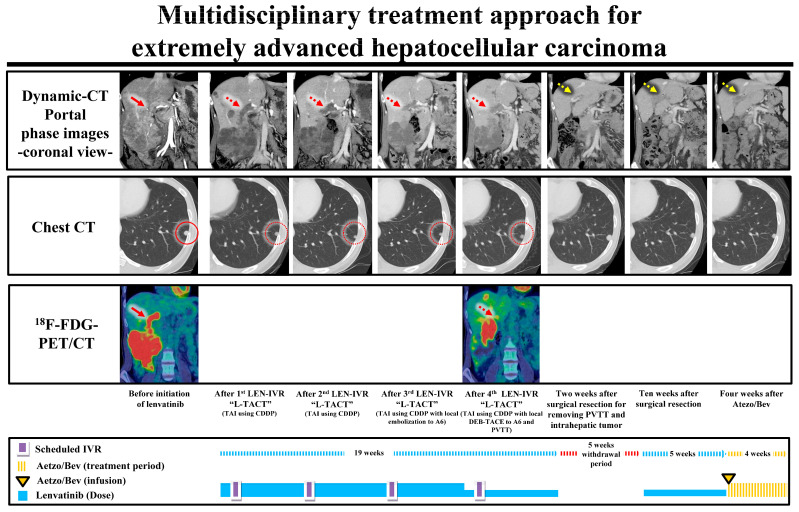
The flow of a multidisciplinary treatment approach for a patient with extremely advanced hepatocellular carcinoma. The patient showed massive portal vein invasion (Vp4) with ^18^F-FDG-PET/CT positive and multiple small lung metastases before the initiation of systemic therapy. We considered the most important prognostic factor was tumor thrombus and, therefore, selected lenvatinib as first-line systemic therapy with the combined use of the “L-TACT” method. After several treatment courses, shrinkage of the tumor was observed, and the tumor thrombus changed from ^18^F-FDG-PET/CT positive to negative. However, the relative dose of lenvatinib was decreased due to its side effects. Therefore, we performed conversion surgery on the liver tumor and tumor thrombus to manage intrahepatic tumor control. However, the recurrence of the tumor thrombus occurred during the withdrawal of lenvatinib. Therefore, we changed systemic therapy from lenvatinib to Atezo/Bev as second-line treatment, which resulted in good control of the tumor thrombus. The size and number of the multiple small lung metastases were also stable during the treatment period. The red arrow shows the tumor thrombus, and the red dotted arrow indicates the time course of the tumor thrombus during lenvatinib-based treatment. The yellow dotted arrow indicates the time course of the recurrence of the tumor thrombus. The red circle indicates lung metastases, and the red dotted circle indicates the time course of the lung metastases. Atezo/Bev—atezolizumab plus bevacizumab; ^18^F-FDG-PET/CT—^18^F-fluorodeoxyglucose positron emission tomography/computed tomography; L-TACT—lenvatinib-transhepatic arterial cisplatin infusion sequential therapy.

Therefore, the most important clinical message from this study is that it is necessary to consider MCT during systemic therapy in order to control intrahepatic target nodules, even in patients with BCLC stage C HCC. As stated in previous reports [15,16,17,18,28,29], lenvatinib-TACE sequential therapy is one of the useful subsequent LTs for controlling intrahepatic target nodules when a patient has intrahepatic target nodules. It is also important to control treatment intensity to maintain sufficient residual liver function when additional subsequent treatment is administered. Moreover, it has been reported that the combined use of lenvatinib and hepatic arterial infusion chemotherapy is effective in advanced HCC and has encouraging survival benefits [38]. In addition, hepatic arterial infusion chemotherapy generally has less effect on hepatic functional reserve. This is also considered to be an essential factor when selecting additional treatment, with the key decision-making factor being intrahepatic tumor burden. Patients where the intrahepatic tumor burden “exceeds the up-to-7 criteria out” indicate a significantly worse prognosis. Therefore, it is regarded as one of the most important factors for predicting overall survival in patients with BCLC stage C HCC treated with systemic therapy. As shown in Figure 8, a multidisciplinary treatment approach may lead to desirable long-time survival for patients with extremely advanced HCC. 

Therefore, it is important to constantly focus on intrahepatic tumor burden during the treatment course of BCLC stage C HCC using systemic therapy and always keep in mind the best time to change to the MCT strategy.

It is important to note that our study had several limitations. These included its retrospective design, single-center setting, and relatively small patient cohort. We also used a variety of subsequent treatments, due mainly to the absence of an established treatment strategy for immune-based therapies. On the other hand, the overall survival is also worse than previously reported data from large clinical trials, as shown in Figure 1 of this study. One reason may be that the study used real clinical data that patients had more varied clinical backgrounds. Therefore, there is more variation in background factors and a larger number of patients with worse disease conditions than in clinical trials. These backgrounds, combined with the limited number of cases in our study, may result in difficulties in determining the impact of each treatment. In addition, the study was based on a limited number of small cases, making it difficult to stratify treatments in more detail. Therefore, further studies using larger multicenter cohorts and longer follow-ups are needed to validate our results. In addition, some patients could not be switched to another systemic therapy and/or LT during subsequent treatment due to decreased hepatic functional reserve that occurred as a consequence of the historical background and clinical management. Therefore, the optimal timing and strategies for sequential treatment should also be considered in future clinical trials.

## 5. Conclusions

MCT is more effective in patients with BCLC stage C HCC and intrahepatic target nodules who have received systemic therapy-based treatment. In addition, the key decision-making factor in these patients is the extent of intrahepatic tumor burden. Patients with an intrahepatic tumor burden that “exceeded the up-to-7 criteria” had a significantly worse prognosis, indicating that these criteria are one of the most important prognostic factors for predicting OS in patients with BCLC stage C HCC treated with systemic therapy. Therefore, it is necessary to constantly focus on intrahepatic tumor burden during the treatment course of BCLC stage C HCC treated with systemic therapy and always keep in mind when to switch to the MCT strategy.

## Figures and Tables

**Table 1:** Clinical profiles and laboratory data of patients with BCLC stage C HCC treated with systemic therapies *.

Patient Characteristics and Laboratory Data	
Number of patients	64
Sex, males:females, n (%)	51 (80%):13 (20%)
Age, yr (range) ^†^	73.5 (41–93)
Body mass index, kg/m^2^ (range)	22.6 (16.4–34.8)
HCV: HBV: nonB, nonC, n (%)	28 (44%):13 (20%):23 (36%)
Performance status 0:1, n (%)	60 (94%):4 (6%)
Platelet count, ×10^3^/μL (range) ^†^	171 (52–371)
Albumin, g/dL (range) ^†^	3.7 (2.9–4.6)
Total bilirubin, mg/dL (range) ^†^	1.0 (0.3–2.1)
Prothrombin activity, % (range) ^†^	86.3 (63.3–109.2)
AST, IU/L (range) ^†^	41.5(15–351)
Neutrophil-to-lymphocyte ratio, % (range)	3.13 (1.35–8.94)
AFP, μg/L (range) ^†^	89.0 (1.5–61,040.7)
DCP, AU/L (range) ^†^	691.0 (12.0–194,798.0)
Child-Pugh score 5:6, n (%)	41 (64%):23 (36%)
mALBI grade (1:2a:2b:3), n (%)	18 (28%):24 (38%):22 (34%):0 (0%)
Lenvatinib: Atezolizumab plus Bevacizumab [n (%)]	45 (70%):19 (30%)
Initial dose of lenvatinib, 4 mg:8 mg:12 mg [n (%)]	4 (9%):18 (40%):23 (51%)
Reduced starting dose of lenvatinib [n (%)]	5 (11%)
**Tumor characteristics**	
Largest tumor diameter, mm (range) ^†^	46.0 (11.0–175.0)
Exceeding the Up-to-7 criteria, n (%)	45 (70%)
Macrovascular invasion, n (%)	39 (61%)
Extrahepatic metastasis, n (%)	38 (59%)
TACE failure/refractoriness, n (%)	29 (45%)

* AFP, alpha-fetoprotein; BCLC, Barcelona Clinic Liver Cancer; AST, aspartate aminotransferase; DCP, des-γ-carboxyprothrombin; HBV, hepatitis B virus; HCC, hepatocellular carcinoma; HCV, hepatitis C virus; IU, international units; mALBI, modified albumin-bilirubin; NonB, NonC, neither HBV nor HCV infection present; NLR, neutrophil-to-lymphocyte ratio; and TACE, transarterial chemoembolization; ^†^ Data expressed as median (range). The ratios are rounded off to the first decimal place and therefore the total will not necessarily be 100.

**Table 2:** Predictive factors for overall survival in patients with BCLC stage C HCC treated with systemic therapy.

	*p* *	Coefficients ^†^	SE	Wald χ^2^	HR	95% CI
Exceeding the Up-to-7 criteria	0.011	1.112	0.437	6.480	3.040	1.291–7.155
AFP +100 µg/L	0.023	0.003	0.001	5.198	1.003	1.000–1.006
DCP +100 AU/L	0.006	0.001	0.000	7.587	1.001	1.000–1.002
**Subsequent treatment during treatment period**						
No subsequent treatment						
Single treatment procedure	0.003	−1.276	0.429	8.862	0.279	0.120–0.647
Multidisciplinary combination therapy	<0.001	−1.667	0.448	13.838	0.189	0.078–0.454
R0 resection	0.006	−2.932	1.077	7.412	0.053	0.006–0.440

* Based on the likelihood test adjusted for the other factors in the final model. ^†^ Estimated coefficient for the variable and the associated standard error. Abbreviations: AFP, alpha-fetoprotein; 95% CI, 95% confidence interval; DCP, des-γ-carboxyprothrombin; HR, hazard ratio; mALBI, modified albumin-bilirubin; SE, standard error; and TACE, transarterial chemoembolization. *Note.* Multivariate Cox regression was applied using stepwise backward selection. Of the potential predictors, factors showing a marginal association (*p* < 0.15) with overall survival after the introduction of lenvatinib in the univariate analysis were included in the initial model. Factors that showed no or limited statistically significant association (*p* > 0.1) were then adjusted for the remaining factors in the model and deleted from the model in a stepwise fashion. The following 13 variables were tested (*p*-values in univariate analysis): age (0.130), sex (0.046), body mass index (0.016), etiology (non-viral vs. viral) (0.554), mALBI grade (1, 0.008; 2a, 0.024; and 2b, 0.002), serum a-fetoprotein (<0.001), plasma des-γ carboxyprothrombin (0.008), Up-to-7 criteria (within vs. exceeding) (0.030), macrovascular invasion (0.068), extrahepatic metastasis (0.272), neutrophil-to-lymphocyte ratio (0.079), TACE failure/refractoriness (0.559), and type of subsequent treatment during treatment period (single treatment procedure, 0.058; multidisciplinary combination therapy, 0.006; and R0 resection, 0.008).

**Table 3:** Clinical features of patients with HCC treated with systemic therapy followed by with or without subsequent treatment *.

	None	STP	MCT	R0	*p*-Value
	n = 22	n = 19	n = 19	n = 4	
**Pretreatment patient characteristics**					
Age, yr (range) ^†^	77.5 (45–93)	79.0 (55–88)	70.0 (41–86)	68.5 (64–70)	0.039
Viral:Non-viral, n (%)	14 (64%):8 (36%)	12 (63%):7 (37%)	14 (74%):5 (26%)	1 (25%):3 (75%)	0.371
mALBI grade (1:2a:2b), n (%)	2 (9%):10 (45%):10 (45%)	5 (26%):8 (42%):6 (32%)	8 (42%):5 (26%):6 (32%)	3 (75%):1 (25%):0 (0%)	0.091
AFP, µg/L (range) ^†^	144.0 (1.5–61,040.7)	121.1 (3.0–55,372.0)	28.3 (3.0–18,861.6)	10.7 (4.9–87.0)	0.143
DCP, AU/L (range) ^†^	1628.5 (16.0–63,347.0)	294.0 (12.0–116,157.0)	483.0 (18.0–194,798.0)	1671.0 (175.0–3608.0)	0.502
NLR, % (range) ^†^	3.48 (1.35–6.92)	2.76 (1.36–6.08)	3.27 (1.89–8.94)	2.18 (1.56–2.62)	0.101
Lenvatinib:Atezo plus Bev [n (%)]	16 (73%):6 (27%)	15 (79%):4 (21%)	10 (53%):9 (47%)	4 (100%):0 (0%)	0.188
**Pretreatment tumor characteristics**					
Largest tumor diameter, mm (range) ^†^	50.9 (11–175)	46.0 (11–116)	46.0 (17–108)	36.5 (22–54)	0.774
Exceeding the Up-to-7 criteria, n (%)	13 (59%)	15 (79%)	16 (84%)	1 (25%)	0.055
Macrovascular invasion, n (%)	11 (50%)	14 (74%)	12 (63%)	2 (50%)	0.453
Extrahepatic metastasis, n (%)	15 (68%)	11 (58%)	11 (58%)	2 (50%)	0.723
TACE failure/refractoriness, n (%)	12 (55%)	9 (47%)	7 (37%)	1 (25%)	0.641

* AFP, alpha-fetoprotein; Atezo plus Bev, atezolizumab plus bevacizumab; DCP, des-γ-carboxyprothrombin; HCC, hepatocellular carcinoma; mALBI, modified albumin-bilirubin; MCT, multidisciplinary combination therapy; NLR, neutrophil-to-lymphocyte ratio; STP, single treatment procedure; and TACE, transarterial chemoembolization. ^†^ Data expressed as the median (range). The composition ratio is rounded off to the first decimal place and therefore the total will not necessarily be 100.

## Data Availability

All data generated or analyzed during this study are included in this article. Further inquiries can be directed to the corresponding author.
